# Dose dependent effects of platelet derived chondroitinsulfate A on the binding of CCL5 to endothelial cells

**DOI:** 10.1186/1471-2172-9-72

**Published:** 2008-12-10

**Authors:** Christian Weingart, Peter J Nelson, Bernhard K Krämer, Matthias Mack

**Affiliations:** 1 Department of Internal Medicine II, University of Regensburg, Franz-Josef-Strauss Allee 11, 93042 Regensburg, Germany; 2 Medical Policlinic, University of Munich, Pettenkoferstr. 8a, 80336 Munich, Germany; 3 Department of Internal Medicine I, University of Bochum, Hölkeskampring 40, 44625 Herne, Germany

## Abstract

**Background:**

Chemokines immobilized on endothelial cells play a central role in the induced firm adhesion and transendothelial migration of leukocytes. Activation of platelets at sites of vascular injury is considered to support leukocyte adhesion and extravasation. However, activated platelets also secrete soluble glycosaminoglycans that can interfere with immobilization of chemokines. We therefore analyzed the impact of platelet derived glycosaminoglycans on the immobilization of the chemokine CCL5 (RANTES) on human microvascular endothelial cells and their influence on CCL5-CCR5 interactions.

**Results:**

We confirm that undiluted serum in contrast to plasma decreases binding of CCL5 to endothelial cells. However, when lower concentrations of serum were used, CCL5-presentation on endothelial cells was markedly enhanced. This enhancement was neutralized if serum was digested with chondroinitase ABC. Using different chondroitinsulfate-subtypes we demonstrate that chondroitinsulfate A mediates the enhanced presentation of CCL5 on endothelial cells, whereas chondroitinsulfate B/C even at low concentrations block CCL5 binding. CCR5 downregulation on CCR5-transfected CHO cells or human monocytes is increased by preincubation of CCL5 with serum or chondroitinsulfate A.

**Conclusion:**

We show that chondroitinsulfate A released from platelets increases the binding of chemokines to endothelial cells and supports receptor internalization in a dose dependent manner. These data help to understand the proinflammatory effects of activated platelets.

## Background

The adhesion and transendothelial migration of leukocytes is largely dependent on chemokines and adhesion molecules. In order to support leukocyte recruitment chemokines need to be immobilized on the luminal surface of the endothelial cell wall. Within tissues leukocytes are also directed by gradients of chemokines [1]. By interacting with different chemokine receptors (CCR1 and CCR5) the chemokine CCL5 (RANTES) has been shown to be involved in several steps of leukocyte recruitment [2].

Chemokines can gain access to the luminal site of the endothelium by transcytosis through endothelial cells [3], after release from circulating leukocytes or after secretion from activated endothelial cells [4]. Platelets have been identified as important source of chemoattractant factors such as CCL5 [5], but also release substantial amounts of chondroitinsulfate A [6]. In vivo, platelet activation and adhesion occurs at sites of vascular injury and facilitates leukocyte recruitment [7-10].

It has been shown that membrane bound glycosaminoglycans are critically involved in immobilization and presentation of chemokines [11-14]. Different patterns of glycosaminoglycan expression on cells may favor the binding of certain chemokines and thereby influence the cellular composition of the inflammatory response. However, chemokines also interact with soluble glycosaminoglycans that compete with the binding of chemokines to cell surfaces. Heparin has the highest affinity to CCL5, followed by heparansulfate, chondroitinsulfate C, dermatansulfate (chondroitinsulfate B) and chondroitinsulfate A [15]. We could demonstrate that human serum inhibits CCL5 binding on CHO cells and cultured human endothelial cells and could identify the responsible serum factor as chondroitinsulfate A (CSA) released from platelets after activation [16].

Glycosaminoglycans also alter the ability of chemokines to interact with chemokine receptors. Soluble Glycosaminoglycans have been shown to inhibit binding of IL-8 to CXCR1 and CXCR2 and CCL3 to CCR1 [15]. It was also shown that CCL5/glycosaminoglycan complexes are able to bind to deglycated PBMC and thereby block HIV-1 infection more effectively than CCL5 alone [17].

Activated platelets have been identified as a major source of CSA in human serum [6]. In addition, release of chondroitinsulfate A was shown in activated T cells [18,19]. It is commonly thought that interaction of chemokines with soluble glycosaminoglycans reduces their ability to bind to cell surfaces and interferes with leukocyte recruitment. However, these results do not fit to the proinflammatory effects caused by intravascular activation of platelets. Therefore we analyzed in more detail the influence of serum and various glycosaminoglycans on the binding of CCL5 to endothelial cells and on the ability of CCL5 to activate CCR5.

## Results and discussion

### Influence of serum and glycosaminoglycans on CCL5 binding to endothelial cells

**Figure 1:**
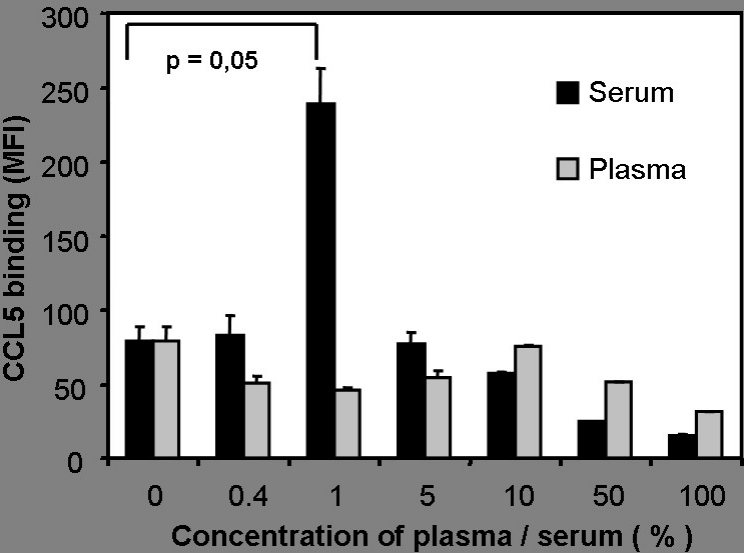
**Influence of human serum and plasma on the binding of CCL5 to endothelial cells**. CCL5 (1 μg/ml) was preincubated with different concentrations of human serum (black bars) or human plasma (grey bars) and subsequently incubated with human microvascular endothelial cells. Dilutions were performed with PBS. Binding of CCL5 to the cell surface was quantified by FACS-analysis and is given as mean fluorescence intensity (MFI). Preincubation of CCL5 with low concentrations of serum significantly enhanced CCL5 binding, while preincubation with high concentrations of serum reduced CCL5 binding. Preincubation of CCL5 with plasma did not result in an increased immobilization of CCL5.

**Figure 2:**
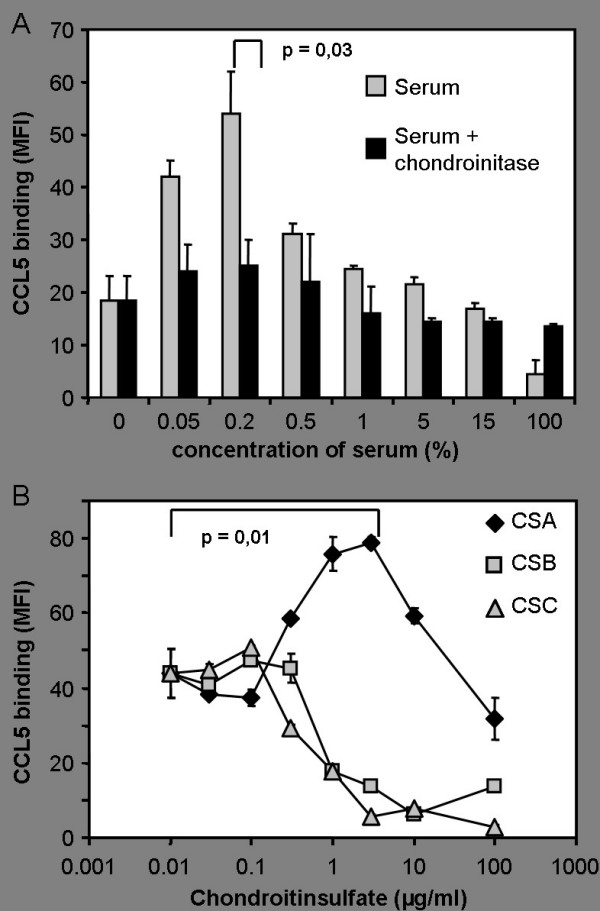
**Chondroitinsulfate A enhances the binding of CCL5 to endothelial cells**. A) Digestion of serum with chondroinitase ABC abrogates the effects of serum on CCL5 binding. Serum was digested with chondroinitase ABC (grey bars) or left undigested (black bars) and then incubated at different concentrations with human CCL5 (1 μg/ml). Subsequently, binding of CCL5 to human microvascular endothelial cells was quantified by FACS-analysis and is given as mean fluorescence intensity (MFI). Undigested serum reduced or increased the binding of CCL5 in a concentration dependent manner, while digested serum did not alter CCL5 binding. Maximal reduction of CCL5 binding reached statistical significance (p = 0.03) compared to undigested serum. B) Influence of various types of chondroitinsulfate on the binding of CCL5 to human microvascular endothelial cells. CCL5 (1 μg/ml) was preincubated with different concentrations of chondroitinsulfate A (CSA), chondroitinsulfate B (CSB) or chondroitinsulfate C (CSC). Preincubation of CCL5 with low concentrations of CSA results in an significantly (p = 0.01) enhanced binding of CCL5 to endothelial cells, while preincubation of CCL5 with high concentrations of CSA or with CSB and CSC results in a reduced binding of CCL5.

In previous experiments we have shown a reduced binding of CCL5 to cell surfaces after preincubation of CCL5 with human serum and have identified the responsible serum factor as chondroitinsulfate A (CSA) released from activated platelets. In these experiments we used undiluted or moderately diluted serum and high concentrations of CSA [16]. The use of undiluted serum or high concentrations of glycosaminoglycans may not reflect in vivo settings of local inflammation where only a minor proportion of total platelets are activated and release CSA. We therefore analyzed the effects of serum or chondroitinsulfate A over a wide range of concentrations. CCL5 was preincubated with decreasing concentrations of human serum and then incubated with microvascular human endothelial cells (MVEC) (Fig. 1). High concentrations of serum block CCL5 binding to the surface of microvascular endothelial cells confirming our previous results [16]. However, preincubation of CCL5 with lower concentrations of serum resulted in a threefold higher binding of CCL5 to endothelial cells (Fig. 1). Human plasma, that does not contain CSA released from activated platelets, was unable to increase the binding of CCL5 to endothelial cells suggesting that platelet derived chondroitinsulfate A might be a crucial factor. To demonstrate that chondroitinsulfate is responsible for the enhanced binding of CCL5 in the presence of serum, serum was digested with chondroinitase ABC before preincubation with CCL5. Digested serum was no longer able to reduce or enhance the binding of CCL5 (Fig. 2A). To determine which glycosaminoglycan is responsible for this effect, we compared the effect of CSA, CSB and CSC on CCL5 binding to endothelial cells (Fig. 2B). Preincubation of CCL5 with CSB and CSC resulted in a strong inhibition of chemokine binding. No increased binding of CCL5 was detectable even at low concentrations of these glycosaminoglycans. In contrast, preincubation of CCL5 with low concentrations of CSA resulted in a dose-dependent increase of the binding of CCL5 (Fig. 2B). The most likely explanation for the enhanced binding of CCL5 after exposure to low concentrations of CSA or serum is the occurrence of CCL5-CSA complexes and their subsequent binding to surface glycosaminoglycans via their CCL5 part or to surface receptors for CSA via their CSA part. Among molecules that are known to bind chondroitinsulfate A (CD36, CD206 or CD44 [20,21]) only the proteoglycan CD44 was strongly expressed on cultured endothelial cells. However, neither blockade of CD44 with a neutralizing antibody (BRIC235 [22]) nor preincubation of cells with serum, CSA, CSB or CSC altered the binding of CCL5 or CCL5-CSA-complexes to the surface of endothelial cells (data not shown). Therefore it seems unlikely that possible CSA binding sites on cell surfaces play an important role in the process of chemokine immobilization. Instead, we favor the hypothesis that CCL5-CSA complexes bind primarily via CCL5 to glycosaminoglycans on the cell surface. The low affinity between CCL5 and CSA may allow the binding of CCL5-CSA complexes to glycosaminoglycans with high affinity for CCL5 (e.g. heparansulfate). Surface heparansulfate proteoglycan expression is upregulated under proinflammatory conditions in osteosarcoma cells [23]. To test the hypothesis that activation of endothelial cells leads to an increased immobilization of chemokines, cells were stimulated with TNF-α and IL-1β for 48 hours. However, stimulation of endothelial cells with TNF-α and IL-1β did not increase CCL5 immobilization on primary human microvascular endothelial cells (data not shown).

We also investigated if CCL5 bound to the surface of endothelial cells could be removed from the cell surface by incubation with an excess of soluble glycosaminoglycans. High doses of CSC (100 μg/ml) in contrast to CSA resulted in a minor reduction (< 20%) of surface bound CCL5 (data not shown).

### Enhanced downregulation of CCR5 by CCL5-CSA complexes

**Figure 3:**
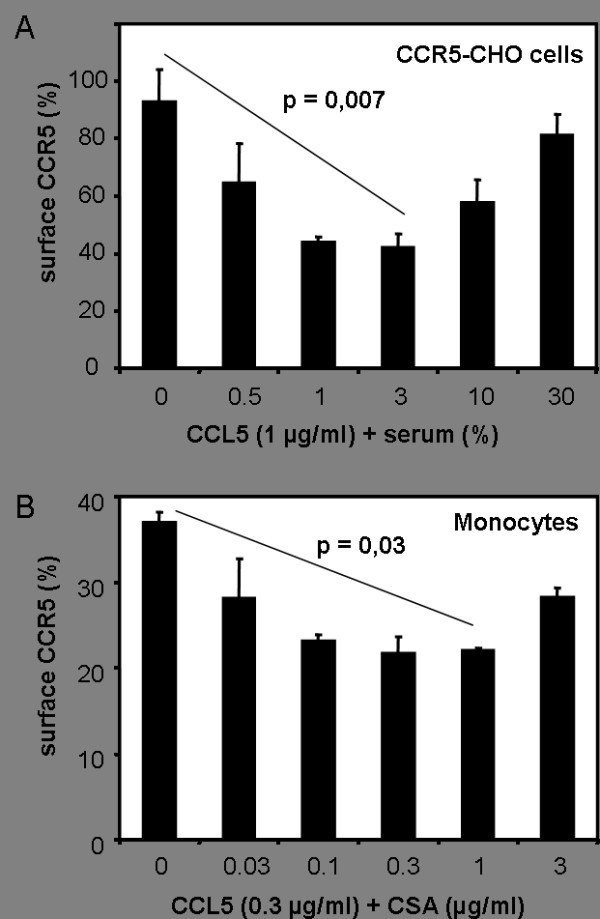
**Enhanced downmodulation of CCR5 by preincubation of CCL5 with serum or CSA**. A) CCL5 (1 μg/ml) was preincubated with various concentrations of human serum diluted in PBS and then used to downmodulate CCR5 from CCR5-transfected CHO cells. Low concentrations of serum significantly (p = 0.007) enhance the ability of CCL5 to downmodulate CCR5 from the surface of CHO cells. Serum alone did not induce downmodulation of CCR5 from the surface of CHO cells (data not shown). B) CCL5 (0.3 μg/ml) was preincubated with various concentrations of CSA and then used to downmodulate CCR5 from the surface of human monocytes. CSA alone did not affect CCR5 expression on monocytes (data not shown), but increased the CCL5-induced downmodulation of CCR5 significantly (p = 0.03).

To assess whether the interaction of CCL5 with CSA alters the ability of CCL5 to activate CCR5, we performed receptor downmodulation assays with CCR5 transfected CHO cells and human PBMC isolated from peripheral blood [24]. Surface expression of CCR5 was quantified by FACS analysis. As shown in Figure 3A, downregulation of CCR5 on CHO cells was markedly enhanced if CCL5 was preincubated with serum. Serum concentrations of between 1 % and 3 % resulted in the most pronounced enhancement of CCL5 induced CCR5 downregulation compared to CCL5 alone. Serum alone was not able to induce downregulation of CCR5 on transfected CHO cells (data not shown). We also investigated CCL5-induced downmodulation of CCR5 on human PBMC and analyzed CCR5 surface expression on human monocytes by flow cytometry (Fig. 3B). Preincubation of CCL5 with CSA resulted in a more pronounced downmodulation of CCR5 from the cell surface than preincubation of CCL5 with PBS. CSA alone had no significant effect on CCR5 expression on human monocytes. These data demonstrate that complex formation of CCL5 with CSA not only increases the binding of CCL5 to cell surfaces but also enhances the ability to interact with the chemokine receptor CCR5.

## Conclusion

Activation of platelets at sites of vascular injury is considered as an important proinflammatory stimulus. Here we show that chondroitinsulfate A released from activated platelets has pronounced dose dependent effects on the immobilization of CCL5 to endothelial cells and the interaction of CCL5 with CCR5. Only at high concentrations does platelet derived CSA block CCL5 binding to endothelial cells, whereas at lower concentrations of CSA the immobilization of CCL5 and its interaction with CCR5 are markedly enhanced. Our data improve our understanding of how activation of platelets may contribute to an increased recruitment of chemokine receptor expressing leukocytes.

## Methods

### Cells and reagents

Human microvascular endothelial cells (Promocell, Heidelberg, Germany) were cultured in MCDB 131 medium (Invitrogen, Karlsruhe, Germany) with 15% FCS, hydrocortisone (1.2 μg/ml), epidermal growth factor (3 μg/l) and L-glutamine (4 mmol/l). Where indicated endothelial cells were stimulated for 48 h with TNF-α (10 ng/ml) and IL-1β (5 ng/ml) (PeproTech, Rocky Hill, New Jersey USA). CCR5 transfected CHO cells [24] were cultured in nucleotide free alpha-MEM medium containing 10% dialyzed FCS. Human PBMC were isolated from healthy human individuals by Ficoll-Paque density gradients and cultured in RPMI medium (Invitrogen, Karlsruhe, Germany) for 24 hours to induce CCR5 expression on monocytes. Human serum and plasma were obtained from healthy volunteers using standard blood sample equipment. Plasma was anticoagulated with EDTA. Where indicated serum was digested for 2 hours at 37°C with chondroinitase ABC (final concentration of 50 mU/mL; Sigma-Aldrich) in RPMI 1640 medium.

### Binding of CCL5 to cell surfaces

Recombinant CCL5 (PeproTech, Rocky Hill, New Jersey USA) was preincubated for 1 h on ice with PBS or different concentrations of serum, plasma, chondroitinsulfate A (CSA), chondroitinsulfate B (CSB) or chondroitinsulfate C (CSC) (Sigma, St. Louis, USA) as indicated in the figure legends. Cells were then incubated for 1 h on ice with CCL5. After four washing steps with PBS, cells were stained with a monoclonal antibody against CCL5 (VL-1, 10 μg/ml) (kindly provided by Dr. Nelson, Munich, Germany) or a mouse IgG2b isotype control antibody (Sigma-Aldrich, St. Louis, USA) followed by a phycoerythrin-conjugated, rabbit anti-mouse F(ab)2 fragment (DAKO, Glostrup, Denmark). Alternatively staining was performed with a biotinylated antibody against CCL5 (clone VL-1, CALTAG, Burlingame, USA) followed by allophycocyanin-conjugated Streptavidin (BD-Pharmingen, Heidelberg, Germany). CCL5-binding was quantified by flow cytometry using a FACSCalibur and cell quest analysis software and is given as mean fluorescence intensity (MFI) (Becton Dickinson, Heidelberg, Germany).

Experiments to detect or block CD44 were performed with the anti-CD44 clone BRIC 235 (Bristol Institute for Transfusion Sciences, Bristol, UK) at concentrations of 10 and 20 μg/ml respectively. Expression of CD206 (mannose receptor) and CD36 was measured with monoclonal antibodies against CD206 (clone 19.2) and CD36 (clone CB 38) by flow cytometry (BD-Pharmingen, Heidelberg, Germany).

### Downmodulation of CCR5

Downmodulation of CCR5 was performed as described previously with minor modification [24]. CCL5 was preincubated with serum or CSA as described above. CCR5 transfected CHO cells or PBMC were incubated at 37°C for 2 hours with preincubated CCL5 (1 μg/ml) or with PBS (pos. control) to induce receptor downmodulation. Further steps were performed on ice to avoid receptor recycling. CCR5 expression was measured by flow cytometry using the monoclonal antibody MC-1 (10 μg/ml) or an mouse IgG1 isotype control antibody (neg. control), followed by a phycoerythrin-conjugated, rabbit anti-mouse F(ab)2 fragment. Surface expression of CCR5 was calculated as [mean fluorescence intensity (exp.) - mean fluorescence (neg. control)]/[(mean fluorescence intensity (pos. control.) - mean fluorescence (neg. control)].

### Statistics

All experiments were reproduced at least two times and single data points were obtained as mean values of duplicates or triplicates. Error bars indicate the standard deviation. Statistical differences of fluorescence intensities were tested by using Student's t-test where appropriate.

## Conflict of interest

The authors declare that they have no competing interests.

## Authors' contributions

CW carried out most experiments and drafted the manuscript, PJN provided the monoclonal antibody against CCL5 and contributed to the design of the experiments, BKK participated in the design of the study and its coordination. MM conceived of the study and participated in its design and coordination. All authors read and approved the final manuscript.
